# Remarks on the Causes, Pathology and Treatment of the Epidemic “Bowel Complaint” of 1849, Commonly Called Epidemic Dysentery

**Published:** 1849-12

**Authors:** Elam Stimson

**Affiliations:** Provincial Licentiate in Physic, Surgery and Obstetrics, St. George, Canada West


### BUFFALO MEDICAL JOURNAL

AND

### MONTHLY REVIEW.

VOL. 5. DECEMBER, 1849. NO. 7.

### ORIGINAL COMMUNICATIONS.

ART. I.—*Remarks on the Causes, Pathology and Treatment of the Epidemic "Bowel Complaint" of* 1849, *commonly called Epidemic Dysentery.* By ELAM STIMSON, M. D., Provincial Licentiate in Physic, Surgery, and Obstetrics, St. George, Canada West.

For the last three years there has prevailed, in many locations in this province, a disease called, in the common-place language of the country, "*the bowel complaint.*"

As far as I have been able to learn, the profession is unsettled as regards its nature and most appropriate treatment. I therefore take the liberty to forward you my (perhaps imperfect and vague) view of its pathology, in as few words as the nature and importance of the subject will admit, trusting that if no other good is effected by it, it will call forth more attention, and elicit that spirit of enquiry, which the disease, from its anomalous character, and fatal tendency, seems to demand.

By many, if not most practitioners, I believe it has been considered and treated as a *dysentery.* Although it is a disease attended by many symptoms in common with dysentery, such as bloody and mucous stools, griping, tenesmus, &c., yet upon an investigation of all the symptoms and circumstances attending it, I believe it will be found to be more nearly allied to *quotidian ague*, or its complication, *chill fever*, and to consist principally in a congested state of the viscera of the abdomen, rather than in chronic inflammation of the mucous membrane of the intestines, in which dysentery essentially consists.

It usually commences with some chilliness, after several days indisposition—sometimes by a distinct chill—at others, only an increased sensibility to cold—again, in others with a *diarrhœa*, which merges in a flux of mucus and blood, attended with griping and tenesmus—commonly, but little fever attends, and this in occasional hot and transient flashes. There is a collapse preceding fatal terminations, which, in most cases, has a striking similarity to that of cholera.

We will now notice some discrepances between the symptoms of this "bowel complaint," and dysentery.

The flux, griping, &c., have more marked exacerbations—assuming quotidian periodicity. In children, an attack, like the ague, is frequently attended with convulsions.

Fatal terminations are earlier, not giving time for inflammation to terminate in ulceration.

The tenderness of the abdomen, so uniformly present in dysentery, is often (even in severe cases) absent, or, if present, is not uniform during the day, much less so during the disease.

In many cases (in children) the evacuations have a more intimate admixture with black or venous blood.

The tongue has a more soft, oily, or slimy appearance. This peculiar appearance of the tongue, as well as the evacuations, I first noticed a short time previous to, and during the prevalence of the cholera in 1832. I am aware that Elliotson says, that in dysentery the evacuations assume every variety of appearance, yet, previous to the summer of 1832, I never noticed that complete admixture of black blood and mucus in the evacuations.

Cases of the bowel complaint not unfrequently occur, that are preceded by intermittent, or chill fever, which, after existing for a time, more or less in different cases, merge in the flux, attended with griping, &c.—while others commence with the flux, griping and tenesmus, and end in intermittent, remittent, or chill fever.

These last facts, alone, might seem sufficient, fully to support the opinion that *the same atmospheric impurity, the malaria, which causes intermittent and chill fever, also causes this "bowel complaint."*

Let us now make an effort to obtain a pathological view of the disease that will cover, as nearly as possible, every organ implicated.

Of the nature of the malaria we can know nothing but by its *effects.* Whether we suppose it some subtle virus which enters and contaminates the blood, by entering it with the oxygen—or, whether it only, by its sedative effect upon the lungs, incapacitates them from the full performance of their excretory function—or, whether we consider (what indeed, is most probable), that in the connection between cause and effect, both conspire, and mutually have the effect to impair the normal condition of the blood and depress the nervous energy of the system, is, in a practical point of view, of little moment.

For the purpose of illustrating the *modus operandi*, and arriving at a comprehensive view of the pathology of the disease, we will suppose the *first* effect of the malaria, is to impair, in some degree, the excretory function of the lungs. A portion of impurity—of the hydrogen and carbon which should be disengaged, passes the lesser, and is sent the round of the general circulation, subjecting every organ and texture to its baleful influence. The vital organs suffering under the depressing influence, or sedative effect of the poison, (for such it is, whether it be matter retained, or has entered the system) become enfeebled in their action. At the same time a degree of inactivity pervades the capillaries, and a *chill*, more or less severe, is the consequence. An undue quantity of blood accumulates in the interior. Ordinarily this accumulation is in the heart and lungs, and their large connecting vessels. But in this "bowel complaint," it falls upon the *abdominal viscera.* From their laxity of texture, and the low degree of vitality of the parts in which the blood vessels are embedded, sufficient reaction does not take place, and the engorged vessels remain unrelieved. They are now in a state of *congestion.* From impaired nervous energy, and the engorged state of the glands, their function also is impaired. For a time they struggle on, and secrete a vitiated product—until, as the disease progresses, a more complete suspension of their functions occur. In accordance with a well known law of the animal economy, the mucous membrane takes on a vicarious action, and the secretion of mucus is excessive. Its acrimony excites pain, and stimulates the peristaltic motion of the intestines, by which it, together with blood oozing from the congested vessels, is expelled from the body.

From the action of impure blood upon the brain—or from deficient nervous energy—or, in more common phrase, by its *sympathising* with the disordered viscera, it becomes disordered in function; also, a most important circumstance, inasmuch as it is liable to give rise to an erroneous opinion that the brain is *inflamed*: an opinion, which, if fully acted upon, would be attended with danger the most imminent. The appropriate treatment being the reverse of phrenitis. But it is the function of the liver and other glands of the abdomen, that are so *uniformly* affected by the malaria. If, after an attack, the evacuations, for a time, continue to show some admixture of bile, it is of a vitiated quality; and when the disease is further advanced, and assumes a more threatening aspect, when the evacuations are little or nothing but a mixture of blood and mucus, there is no evidence of any glandular product.

The secretory function of the abdominal glands are in fact *suspended.*

We may now take a comprehensive view of the whole mischief, as it exists within the abdominal cavity:—

1st. A diminution of nervous power.

2d. The function of the glands impaired or suspended.

3d. The blood vessels congested.

4th. The mucous secretion vitiated, and excessively increased, and blood exuding from the congested vessels upon the surface of the mucous membrane.

To excite the nervous energy, and glandular secretion, to unload sanguineous congestions, and suppress both the excessive secretion of mucus, and exudation of blood, and allay tormina, are the objects to be first attained. These various, and seemingly adverse indications are, in all ordinary cases, simultaneously answered by the conjoined use of *quinine, calomel and opium.* Whatever excites the nervous energy, goes, in some degree, to fulfil the other indications. The patient having been subjected to the same epidemic constitution of the atmosphere that produces intermittent and chill fever, the same constitutional diathesis pervades the system, and the same general principles are applicable in its treatment. Particular reference, therefore, should be had to its *periodicity.* Although these may be but slightly marked by the more ordinary symptoms, or evident increase of chilliness, yet, there is, almost uniformly, an increase in the evacuations, tormina, &c., and the patient if not actually colder, is more susceptible to it. These should be guarded against by giving freely of quinine and opium, particularly three or four hours previous to the time the patient would be affected by a chill, if the disease was ague of quotidian type.

If the treatment be commenced early in the disease, and between the times the patient suffers most, which is usually late in the afternoon, in the evening, or during the night, a portion composed of 1 gr. quinine, from ½ to 1 gr. opium, and from 4 to 6 of calomel, repeated at the end of 3 hours, will usually have the effect to arrest the disease, at least for a time, taking care to fortify the system by giving quinine and as much opium as will produce sensible effect previous to an increase of the evacuations, which may be expected in twenty-four hours from the first marked invasion. Fecal stools will usually follow, without the use of other cathartics, in the course of twenty-four hours. The treatment should be followed up by the use of quinine, opium and *camphor.*

If the attack is one of more than ordinary severity, or is unyielding to the treatment already recommended, it must be met by a corresponding efficiency of treatment. Besides an increase in the use of the remedies mentioned, both in quantity and frequency of repetition, the use of pure and powerful astringents should be added, and external applications employed. The mustard poultice, or rubbing the abdomen with spirits of turpentine, then covering it with a bag of moistened, hot bran, are some of the best, and should be repeated again and again.

Probably the most important point in the treatment of the disease, and one requiring the exercise of much judgment is, after procuring fecal stools, to determine when, and in what degree, to withdraw the use of medicines. Judging from my own experience, less danger is to be apprehended from continuing them longer than may be strictly necessary, than of stopping too soon.

Several practitioners who have tried the treatment recommended above, consider it as sure to arrest the disease for a time, and to procure fecal stools. But their complaint is, that the disease *returns*; the tormina, the mucous and bloody stools, and if not these, an unceasing diarrhœa, with or without the mucous and blood, and then followed collapse.

This relapse, if so it may be called, is, in my opinion, most certainly prevented by a judicious use of quinia, opium, and the muriated tincture, or subcarbonate of iron; to which, in some cases, it may be necessary to add astringents. Judging from a limited experience, the addition of *iron* is very valuable.

The calomel given, seldom *purges.* It seems to have the desired effect of exciting the glandular secretion, and causing fecal evacuations, without its really *cathartic* operation.

Although the use of cathartics may seem necessary to clear the intestines of morbid matter, the product of vitiated secretions, and though many cases may occur in which they may be not only safe, but useful, yet, generally speaking, their use is inadmissible, inasmuch as they occasion an increased determination of blood to the viscera, from which it is all important it should be expelled.

Objectors to the use of any, and every thing that excites, or in any way irritates the mucous surface, should remember, that in reference to the pathological state of the viscera, there is all difference between the stimulus of a cathartic and that of quinine and astringents. *Cathartics* stimulate the mucous follicles of the intestines, and cause a *flux of blood to the parts already gorged,* while quinine, and astringents, &c., stimulate the nervous power, and occasion that action which expels the encumbrance from the parts implicated.

If our premises with regard to the *cause* of this disease, be correct, (malaria) it may, in truth, be said to be chill fever, located in the bowels; the textures, as already said, are lax, possessing a low degree of vitality; little power of resisting an undue influx of blood, and, consequently, there is a non-development of the disease; while the more prominent symptoms, the *evacuations,* having been more observed, and has had the effect to lure the practitioner from a right train of reflection, as regards its nature and cure.

I will only add, that, in my opinion, this epidemic constitution of the atmosphere has an influence upon several diseases *apparently* unconnected, and uninfluenced by it; and should the profession bestow upon it a proper degree of attention, a great service might thereby be rendered.

OCTOBER, 12th, 1849.

